# Insights into Changing Dermatophyte Spectrum in India Through Analysis of Cumulative 161,245 Cases Between 1939 and 2021

**DOI:** 10.1007/s11046-023-00720-6

**Published:** 2023-03-28

**Authors:** Pawan Kumar, S. Ramachandran, Shukla Das, S. N. Bhattacharya, Bhupesh Taneja

**Affiliations:** 1 CSIR-Institute of Genomics and Integrative Biology(CSIR-IGIB), New Delhi, 110025 India; 2 Academy of Scientific and Innovative Research (AcSIR), Ghaziabad, 201002 India; 3 UCMS-GTB, Hospital, Dilshad Garden, Delhi, 110095 India; 4 Dr. Babasaheb Ambedkar Medical College and Hospital, Delhi, 110085 India

**Keywords:** Dermatophytosis, Epidemiology, Phylogenomics, Literature-mining

## Abstract

Dermatophytosis is one of the most common superficial infections of the skin affecting nearly one-fifth of the world population at any given time. With nearly 30% of worldwide terbinafine-resistance cases in *Trichophyton mentagrophytes/Trichophyton interdigitale* and *Trichophyton rubrum* reported from India in recent years, there is a significant burden of the emerging drug resistance epidemic on India. Here, we carry out a comprehensive retrospective analysis of dermatophytosis in India using 1038 research articles pertaining to 161,245 cases reported from 1939 to 2021. We find that dermatophytosis is prevalent in all parts of the country despite variable climatic conditions in different regions. Our results show *T. rubrum* as the most prevalent until 2015, with a sudden change in dermatophyte spectrum towards *T. mentagrophytes*/*T. interdigitale* complex since then. We also carried out an 18S rRNA-based phylogenetics and an average nucleotide identity-and single nucleotide polymorphism-based analysis of available whole genomes and find very high relatedness among the prevalent dermatophytes, suggesting geographic specificity. The comprehensive epidemiological and phylogenomics analysis of dermatophytosis in India over the last 80 years, presented here, would help in region-specific prevention, control and treatment of dermatophyte infections, especially considering the large number of emerging resistance cases.

**Supplementary Information:**

The online version contains supplementary material available at 10.1007/s11046-023-00720-6.

## Introduction

Global Burden of diseases lists fungal skin diseases as the fourth most prevalent disease globally with significantly impaired quality of life of the patients [1–3]. Among fungal skin diseases, superficial infection of the skin, hair and nails termed dermatophytosis is the most common fungal infections, affecting 20–25% of the world population at any given time [4–7]. Dermatophytosis is associated with considerable morbidity and socio-economic trauma. An estimated annual economic burden of direct healthcare costs of $2 billion and an additional $1 billion due to lost productivity of the patients is associated with skin fungal (and viral) infections in the US alone [8]. Although estimated data for other countries is not available, equivalent large losses in economy and development are anticipated in other countries as well.

The main causative agents of dermatophytosis are a group of keratinophilic filamentous fungi belonging to the three closely-related genera *Trichophyton, Microsporum* and *Epidermophyton* and collectively termed dermatophytes [9, 10]. Although dermatophytes are found throughout the world, the distribution of strains and the most common sites of infection vary with climatic, geographic, socioeconomic and prevalent lifestyle conditions in the respective regions [4, 11, 12]. For instance, *Trichophyton rubrum* is the predominant dermatophyte in clinical isolates from central and north Europe [4, 13] whereas *T. mentagrophytes* is more commonly reported from Asia [11, 14]. Zoophilic dermatophytes, *Microsporum canis* or *Trichophyton verrucosum*, on the other hand, are frequently reported from Southern Europe and the middle East countries [11].

Apart from the prevalent geographic specificity of the causative agent, dermatophytes have often exhibited a unique phenomena of a significant shift in prevalent dermatophyte spectrum in several countries over different periods of time [11]. For instance, in Germany, the shift to *T. rubrum* as the most prevalent dermatophyte (causing 70–80% of all cases by 1990s), was preceded by only 40% *T. rubrum* infections in the 1950s and *Epidermophyton floccosum* and *Microsporum audouinii* as predominant dermatophytes (~ 70% of all representative cases) in the 1920s. In China, a continuous shift in dermatophyte spectrum has been observed; from *T. rubrum* upto 1950s to *T. schoenleinii* and *T. violaceum* in 1980s to zoophilic *Microsporum canis* in tinea capitis infections since then [15, 16]. Similarly, ~ 75% of *T. rubrum* cases in 1990s in Slovakia was preceded by only 25% of reported *T. rubrum* infections in 1950s, replacing the zoophilic dermatophytes *Trichophyton verrucosum* and *Trichophyton mentagrophytes* (55% to 10% cases from 1950 to 1990s) as the primary dermatophyte agents. In USA, a decline in *T. rubrum* cases from 53.7 (in 1979–1981) to 41.3% (in 1993–1995) had a concordant increased frequency of *T. tonsurans* cases from 27.9 to 44.9% in the corresponding period [11]. Comparison of the dermatophyte spectrum hence reflects the epidemiological situation of dermatophytosis in the respective country that appears to change over a period of time.

In India, there are an increasing number of dermatophytosis reports in the last 10–12 years (for instance, [17–25]). In addition, there seems to be a shift in predominant causative organism with increasing reports of *T. mentagrophytes* as a primary pathogen in a few recent studies [26]. Moreover, an 18S rRNA-based phylogenetic analysis of the major pathogenic strains from India shows that the pan-Indian strains of *T. mentagrophytes/ T. interdigitale* complex form a separate clade, termed as genotype VIII, highlighting their unique geographical identity [27, 28]. A congruent increased reports of emerging resistance to antifungal agents [21, 22, 25, 29, 30] emphasizes the need for a detailed epidemiological analysis of prevalent cases and infectious agents in India. A comprehensive study of dermatophytes from India would not only help in terms of (i) proper disease management, (ii) correlate clinical response with geographical specificity of the strains and (iii) help monitor disease progressivity. In this work, through a retrospective analysis, we report the epidemiological study of all reported dermatophytosis cases in the last eight decades in India, from 1939 to 2021. We also carry out an 18S rRNA-based phylogenetics and a whole-genome based phylogenomics study to assess the overall relatedness of prevalent strains in India. To our knowledge, this is a first comprehensive study of epidemiological trends for fungal infections in India that screens more than 1000 articles and > 160,000 cases over a period of 80 years and provides useful information for a region-specific prevention, control and treatment of dermatophyte infections.

## Materials and Methods

### Collection of Data

Collection of data towards prevalence of dermatophytosis in India was carried out using a literature mining approach using PubMed with following MeSH terms (‘*Trichophyton*’ or ‘*Microsporum*’ or ‘*Epidermophyton*’ or ‘tinea’ or ‘dermatophyte’ or ‘onychomycosis’ and ‘India’) along with a filter on publication date upto 30th June, 2021. All shortlisted articles were confirmed for infections in human (and not animal) host before extraction of patient and/or epidemiological data. All review articles, animal studies, description or development of methods (e.g. dermoscopy, trichoscopy, PCR fingerprinting using GACA4 etc.), protein/DNA/RNA estimations, ex vivo/in vivo/cellular studies, immunological studies or drug screening with ATCC strains without description of new isolates, were excluded. Studies with exploratory drugs and random controlled trials (RCT) where no species distribution of causative agents was given and any other article where epidemiological, demographic and topographic details were not provided, were also excluded.

The articles shortlisted with above criteria were then screened manually and relevant epidemiological data such as geographical location of patient or disease, number of reported cases, etiological agents, site of lesions on the host, age, gender, reporting year, annual prevalence, etc. was extracted for further detailed analysis. Sixteen additional articles with inclusion/ exclusion criteria defined above were also identified manually using the bibliography and citation list of shortlisted articles and included for final analysis (Table S1). The compiled data was analyzed with Graphpad Prism v6.01 and complex heatmap module of R v4.1.1.

### Internal Transcribed Spacer (ITS)-Based Phylogenetic Analysis

For phylogenetic analysis, nuclear ribosomal internal transcribed spacer (ITS) regions 1 to 2 of 18S rRNA for dermatophyte isolates from India were downloaded from GenBank (Release 244). Incomplete/partial ITS sequences or sequences containing non-standard (Y, W etc.) or unambiguous nucleotides (N) were not considered. An ITS-based maximum likelihood phylogenetic tree for *T. rubrum, T. mentagrophytes*/ *T. interdigitale* species complex, *T. tonsurans* or *M. gypseum* was constructed using sequences that contained both ITS1 and ITS2 and corresponded to minimum lengths of 554 bp, 590 bp, 577 bp or 460 bp, for each respective organism, using MEGA6 [31] with one thousand bootstrap replicates. For *T. mentagrophytes*/ *T. interdigitale* species complex, all (I to IX) genotypes, as defined by Nenoff et al.[27] were considered for the phylogenetic analysis. *T. indotineae*, recently reannotated from *T. mentagrophytes*/ *T. interdigitale* species complex genotype VIII [32, 33] was considered as *T. mentagrophytes*/ *T. interdigitale* species complex member for construction of the phylogenetic tree.

### Average Nucleotide Identity (ANI)- and Single Nucleotide Polymorphism (SNP)-Based Phylogenomic Analysis

For phylogenomics analysis through overall relatedness of the genomes from India against those from other geographical locations, average nucleotide identity (ANI)-based distance analysis with ANI/AAI-matrix calculator (http://enve-omics.ce.gatech.edu/g-matrix/) [34] and SNP density-based analysis with ParSNP (Harvest suite v1.1.2) [35] were used. The ANI-matrix calculator assesses relatedness on the principle of in silico "DNA hybridization", i.e. sequence conservation for in silico generated ~ 1 kb nucleotide fragments of the entire genomes for a given pair of genomes; higher the ANI, higher the relatedness, with genomes with > 95% ANI classified as same species. ParSNP generates maximal unique matches (MUM) to assess phylogenetic relatedness. Both ANI and ParSNP require assembled genomes as input data.

For whole genome sequence based phylogenomics analysis, nineteen whole genome sequence (wgs) assemblies that were available for *T. rubrum* in GenBank Release 245 (including one genome from India) were downloaded (Table S2). Raw sequence data for additional worldwide genomes from China were available in SRA [36]. However, as our analysis strategy using ANI and ParSNP requires assembled genome sequences, the raw sequence reads could not be carried forward for phylogenomics analysis. For T*. mentagrophytes/ T. interdigitale,* ten genome assemblies (including three from India) (Table S3) were downloaded. Although whole genomes of additional *T. mentagrophytes/ T. interdigitale* genomes from India have been described recently [37], their genome assemblies were not available across genomic resources of EMBL/GenBank/DDBJ and hence phylogenomics of *T. mentagrophytes/ T. interdigitale* was limited to the available ten wgs mentioned above.

For ANI-based phylogenomics for *T. rubrum* or *T. mentagrophytes/ T. interdigitale*, genome-based distance matrix for the respective genome assemblies was obtained and the ANI-dependent distance matrices representing whole genome based phylogeny were plotted. For SNP-based phylogenomics analysis of *T. rubrum* or *T. mentagrophytes*/ *T. interdigitale* genomes from India and rest of the world, SNPs/indel and structural variations were estimated against *T. rubrum* CBS 118892 (CBS 118892) or *T. mentagrophytes* MR816 (MR816) reference genomes, with ParSNP, visualized with Gingr (Harvest suite) and the phylogenetic tree was plotted using iTOL (https://itol.embl.de/).

### Sequence Analysis

Annotated sequences of Erg1 (NCBI Accession ID: XP_003233845.1), Erg11 (NCBI Accession ID: XP_003236980.1) and β -tubulin (NCBI Accession ID: XP_003231604.1) were obtained for CBS 118892 from NCBI. To identify orthologs of these molecular targets in other genomes, all genome assemblies were downloaded from NCBI and structural and functional annotations were carried out using web-interface of AUGUSTUS [38] and PANNZER2 [39] (Protein ANNotation with Z-scoRE), as described earlier [40]. The respective protein orthologs were obtained by carrying out a genome-wide BLAST search in the annotated genomes. Mutations with respect to CBS 118892 and MR816 were identified by aligning the sequences with MEGA6 [31].

## Results

### Prevalence and Clinical Details of Patients

A search in PubMed Central (PMC) with a defined set of keywords associated with dermatophytes and/or dermatophytosis along with an India “constraint” returned one thousand thirty eight articles. All articles were read thoroughly and shortlisted as per inclusion and exclusion criteria defined in the Methods section. Twenty five articles were not available for full text access and had to be rejected. Finally, a total of 330 articles from 1939 to 2021 became available for further evaluation and data extraction for detailed analysis (Table S1). These 330 articles represent reported cases for a total of 161,245 patients with superficial infections in the last eight decades. Of the 161,245 total cases, 16,657 were reported as culture positive for dermatophytes and were considered for further detailed analysis. There is a near homogenous spread of 8,417/16,657 dermatophytosis cases across different decades between 1939 and 2010. However, a sharp increase in the reported cases was observed from 2011 to 2021 (Fig. S1) with nearly 50% of evaluated culture-positive cases (8,240/16,657) reported in this decade alone. This is commensurate with the higher number of 206/330 publications in that decade (Fig. S1).

**Fig. 1:**
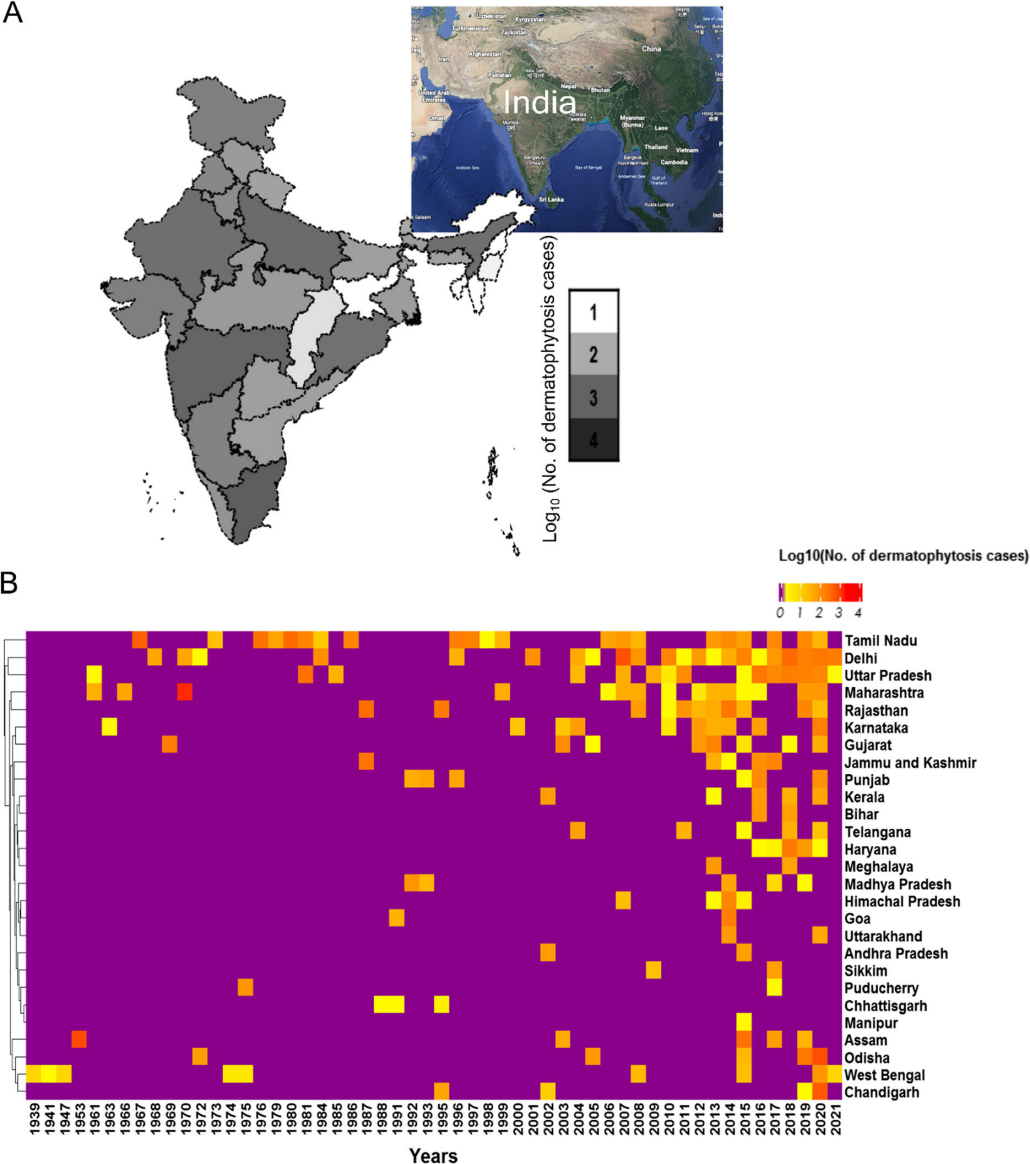
Prevalence of dermatophytosis in India. **A** Distribution of dermatophytosis as reported from different parts of India. A gradient scale indicates Log_10_(number of dermatophytosis cases) in each state from 1939 to 2021. **B** Heatmap of annual incidences of dermatophytosis cases in indicated states from 1939 to 2021. There is intermittent reporting from different states upto 2010. A large increase in dermatophytosis reports is seen subsequently in several states, irrespective of climatic or geographic conditions of those states

Geographical features of India that provide the necessary environmental niche favorable for dermatophyte infections were presumed to have a likely higher prevalence. However, evaluation of reported cases revealed that higher prevalence of dermatophytosis was not limited to any particular location but was reported from several states with different geographical and environmental niches (Fig. 1a). The top five states with highest incidences (Tamil Nadu in south with 2608 cases: coastal, humid weather), (Delhi and Uttar Pradesh in north with 2601 and 1447 cases, respectively: tropical weather), (Maharashtra in the west with 1845 cases: coastal, humid weather and Rajasthan in the west with 1195 cases: dry heat) represent divergent climatic and environmental conditions, from different parts of the country (Fig. 1b). Interestingly, from 1939 to 2010, there are only sporadic reports of dermatophytosis from different parts of India. From 2011 onwards, however, a sudden increase in reports from multiple tertiary care centres is observed, irrespective of previous reporting history or variable climatic or geographic conditions of those regions (Fig. 1a and 1b).

### Changing Dermatophyte Spectrum in India

To analyze the predominant aetiological agents in India across the years and any possible change in their spread with the increasing number of cases, species distribution of dermatophytes was analyzed.

**Fig. 2:**
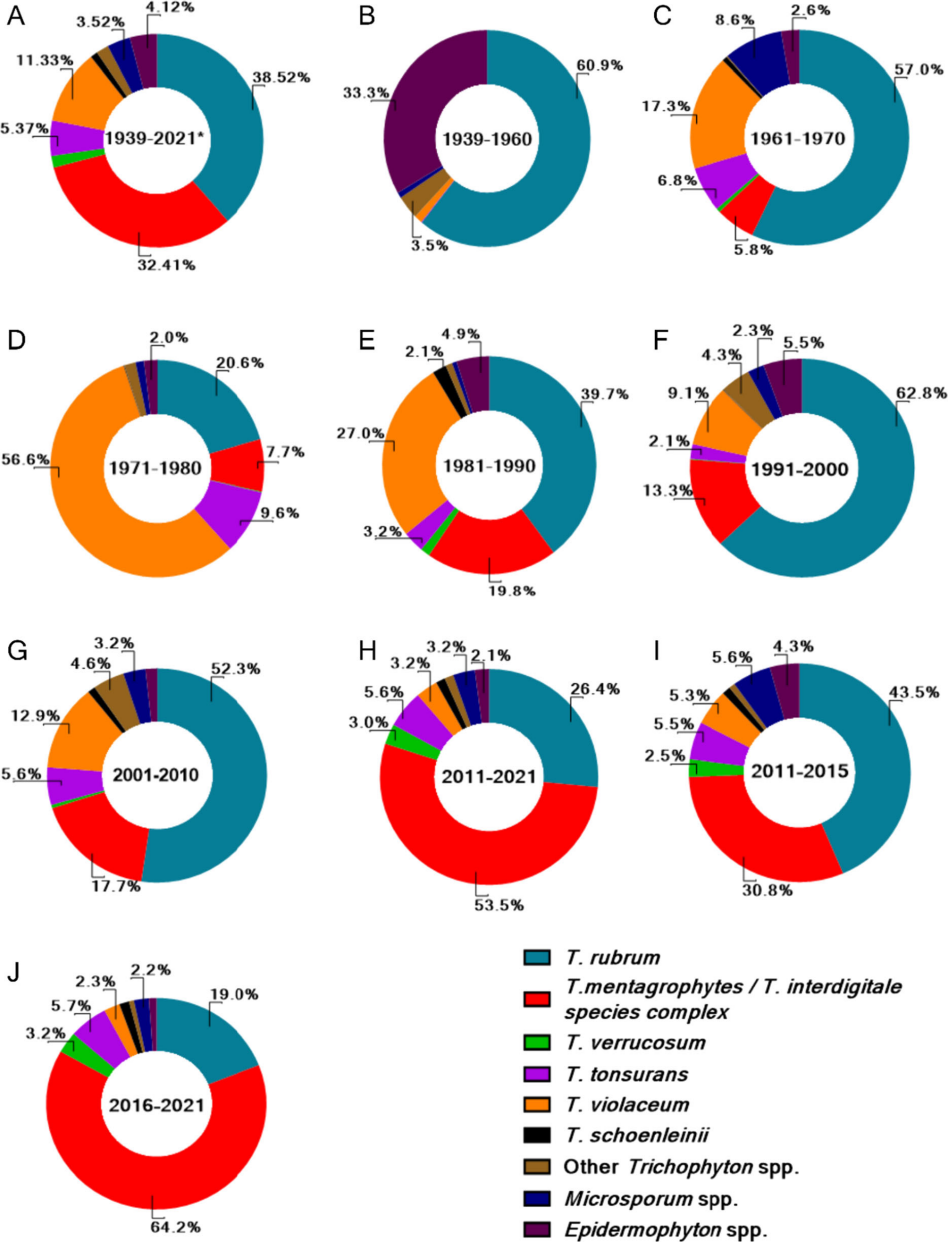
Prevalence of dermatophyte agents in India from 1939 to 2021. Prevalence of predominant aetiological agents in India across the years is plotted. **A** Overall data from 1939 to 2021. **B–H** are decade-wise plots. (**I**) and (**J**) are plots for 2011–2015 and 2016–2021, respectively

Overall, *T. rubrum* is the most common pathogen with 38.5% of total cases between 1939 and 2021 followed by *T. mentagrophytes/ T. interdigitale* complex (32.4%) (Fig. 2a). Further decade-wise analysis indicates that the distribution of prevalent dermatophytes is not constant and has varied over the years. In the initial period of analysis, i.e., 1939–1960, *T. rubrum* is the predominant strain in more than 60% of all reported cases, followed by *Epidermophyton* spp. (33.3%) (Fig. 2b). *T. rubrum* continues to be the most reported pathogen in the following decades, from 1961 to 2010 except 1971–1980 (Fig. 2c-g). In 1971–80, *T. violaceum* is the predominant pathogen with 56.6% of all reported cases followed by *T. rubrum* (20.6%), *T. tonsurans* (9.6%) and *T. mentagrophytes/ T. interdigitale* complex (7.7%) (Fig. 2d).

In 2011–2021, however, there is a dramatic shift in the dermatophyte spectrum, with infections by *T. mentagrophytes/ T. interdigitale* complex (53.3% cases) outnumbering the infections by *T. rubrum* (26.4% cases) or other dermatophytes (Fig. 2h). A closer examination of this changing spectrum was carried out by further breakdown of this analyzed time period into 2011–15 and 2016–2021. While the period 2011 to 2015 itself shows an increase in cases of *T. mentagrophytes/ T. interdigitale* complex with a higher fraction of reported cases (30.8%) as compared to previous decades (Fig. 2i); the shift appears to be more recent as more than 64% of all reported cases in 2016–2021 were ascribed to *T. mentagrophytes/ T. interdigitale* complex (Fig. 2j).

Nevertheless, overall, *T. rubrum* and *T. mentagrophytes/ T. interdigitale* complex are the most prevalent dermatophyte pathogens in India and are together responsible for more than 70% of total infections reported between 1939–2021.

### Topographical Distribution

Whether the change in dermatophyte spectrum is associated with change in type of infections, topographical distribution was next examined in the reported cases. Cumulative analysis of dermatophyte cases across the analyzed period from 1939 to 2021 indicates that tinea corporis was the most prominent infection in India (32.4%), followed by tinea cruris (19.7%), tinea unguium (17.9%), tinea capitis (13.3%), tinea pedis (4.1%), tinea manuum (1.9%), tinea faciei (1.9%), tinea barbae (0.7%) and other lesions (Fig. S2). Tinea corporis, tinea unguium and tinea capitis are the predominant infections across the analyzed time periods with no clear correlation in the trend of clinical manifestations with prevalent pathogens across different decades, possibly due to association of the predominant pathogens, namely, *T. rubrum* and *T. mentagrophytes/ T. interdigitale* complex with multiple types of lesions [41]. In the periods 1961–1970, 1971–1980 and 1981–1990, however, the high number of tinea capitis cases (22.7%, 36.7% and 45.3%, respectively) appears to correlate with the higher infections with *T. violaceum* in those years (Figs. 2c–e and S2).

### Gender Distribution

Among the 176/330 reports where gender information was available for 24,910 patients, the overall distribution of dermatophytosis was 57.2% (14,245/24,910) in adult males and 29.9% (7,440/24,910) in adult females at an adult male: female ratio of 1.91: 1 (Table 1). The adult gender distribution remained skewed towards males in the decades of 1961–70 (M:F: 4.93:1), 1971–80 (M:F: 2.2:1), 1991–2000 (M:F: 3.06:1), 2001–2010 (M:F: 1.45:1) and 2011–2021 (M:F: 1.77:1). A slightly higher prevalence of women with dermatophytosis was observed in the period of 1981–1990 (0.79:1) only, although this difference was not found to be significant (*p*-value = 0.032). Infection in children constituted 3,225 of the total 24,910 reported cases (12.9%) and remained low overall in all the analyzed time frames from 1939–2021. The fraction of cases in children was highest between 1961–1970 and 1981–1990 which corresponded with higher number of reported tinea capitis and *T. violaceum* cases.

**Table 1:** Gender distribution

Years	Male	Female	Child	*p*-value (Male:Female)
1939–1960	–	–	8	–
1961–1970	69	14	165	< 0.0001
1971–1980	3188	1436	1171	< 0.0001
1981–1990	309	389	322	0.032
1991–2000	2539	830	5	< 0.0001
2001–2010	1434	991	153	< 0.0001
2011–2021	6706	3780	1401	< 0.0001
Cumulative (1939–2021)	14,245	7440	3225	< 0.0001

Decade-wise gender distribution of patients with dermatophyte infections. Chi-squared test (*p < 0.05, **p < 0.01, ***p < 0.001 and ****p < 0.0001) was applied between male and female patients

### Geographical distribution: a phylogenetic and phylogenomic perspective

**Fig. 3:**
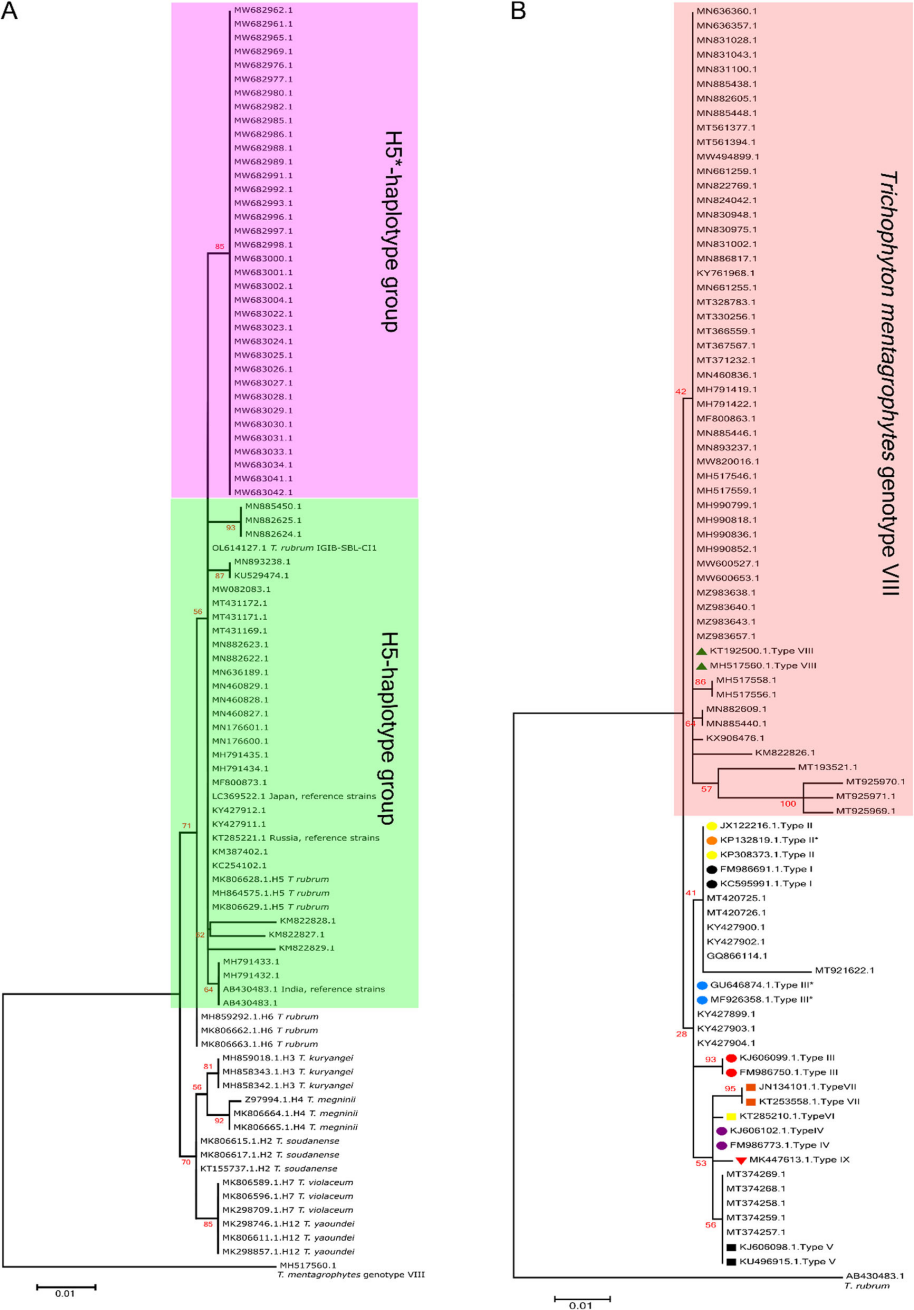
Phylogenetic analysis of *T. rubrum* and *T. mentagrophytes/ T. interdigitale* species complex. 18S rRNA-based maximum likelihood phylogenetic tree with 1000 bootstraps for different pathogens. **A** 18S rRNA-based tree for *T. rubrum*. A H5* sub-group within the H5 haplotype group is formed and indicated. **B** 18S rRNA-based tree for *T. mentagrophytes/ T. interdigitale* species complex. The phylogenetic tree was generated for randomly selected sequences for clarity, while the complete 462 sequences are plotted in Fig. S1. Reference strains for genotypes I to IX are indicated in different colors and form separate clades. As explained in the text, majority of the strains from India group as Genotype VIII. MH517560.1 (*T. mentagrophytes* Genotype VIII), AB430483.1 (*T. rubrum*) were used as outliers in (A) and (B), respectively

With an increasing number of observed dermatophytosis in India, including resistant cases in the last decade, a molecular perspective into the geographical distribution of prevalent pathogens can be obtained through phylogenetic and phylogenomic analysis. We first carried out phylogenetic analysis taking advantage of the large number of 18S rRNA ITS sequences of dermatophytes available in GenBank. For *T. rubrum*, ITS sequences for 66 strains from India were available upto 2021 for the construction of an ITS-based phylogenetic tree. As seen in Fig. 3a, *T. rubrum* strains from India form two major clusters, both corresponding to H5_haplotype as defined earlier [42]. While nearly half of the *T. rubrum* strains (30/66) group together in a cluster containing the reference H5 sequence, the remaining 36/66 sequences form a separate cluster within the H5-haplotype group, which we annotate as H5* to distinguish it from the reference H5 strain.

For *T. mentagrophytes/ T. interdigitale* complex, the ITS-based phylogenetic tree with 462 available ITS sequences for India shows that nearly all strains (449/462) predominantly cluster together with the reference genotype VIII defined earlier [27, 28], in a separate branch from other *T. mentagrophytes/ T. interdigitale* complex genotypes of other geographical locations across the world (Figs. 3b and S3). Only a small subset of sequences from India clustered with genotype I/II/II* (5/462 sequences) or with genotype III* (3/462 sequences) or with genotype V (5/462 sequences) and could possibly be foreign travel-related or zoonotic transfer cases.

**Fig. 4:**
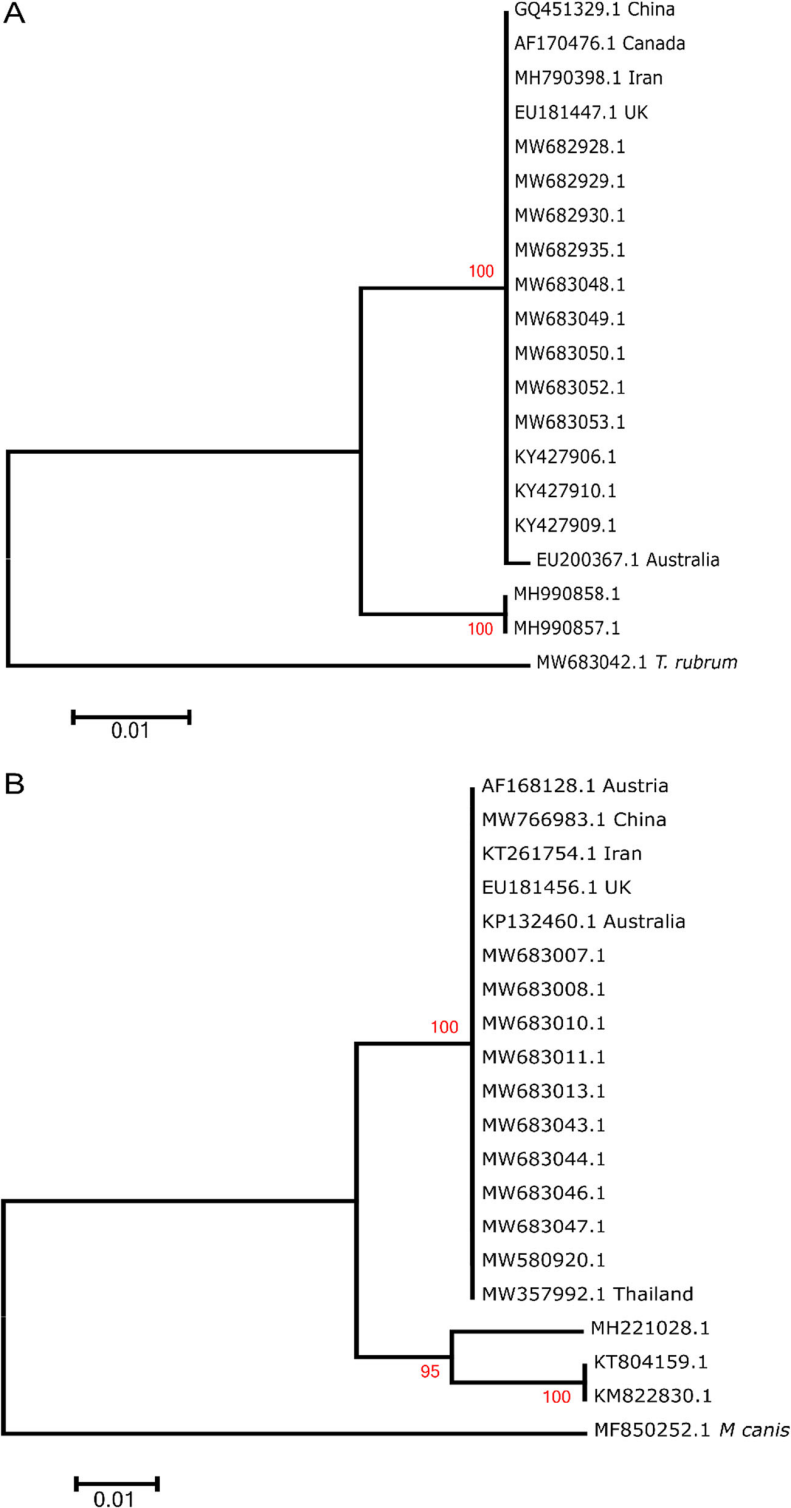
Phylogenetic trees for *T. tonsurans* and *M. gypseum* strains from India. An 18S rRNA-based maximum likelihood phylogenetic tree for **A** *T. tonsurans* and **B** *M. gypseum* isolates from India was generated with 1000 bootstrap replicates. MW683042.1 (*T. rubrum*) and MF850252.1 (*M. canis*) were used as outliers in the trees in (**A**) and (**B**), respectively

The number of ITS sequences in the databases for *T. violaceum* (only five), *E. floccosum* (eight) and *M. canis* (eight), the other predominant pathogens, were insufficient for their phylogenetic analysis. Among the other prominent pathogens identified in India (Fig. 2a), ITS-based phylogenetic trees for *T. tonsurans* (Fig. 4a) and *M. gypseum* (Fig. 4b) could be generated. For both these pathogens, one major phylogenetic cluster was formed that included representative ITS sequences from other parts of the world, suggesting close relatedness among the strains.

ITS-based phylogenetic analysis for dermatophytes hence suggests that ITS sequences are capable of distinguishing geographical specificity of *T. mentagrophytes/ T. interdigitale* complex (genotype VIII) and *T. rubrum* (haplotype H5 and H5*) strains from India through separate clusters or clades in the respective phylogenetic trees. Larger sample sets of ITS sequences are needed for other strains to delineate any geographic or host specificity among them.

### ANI- and SNP-Based Phylogenomics of *Trichophyton* spp.

**Fig. 5:**
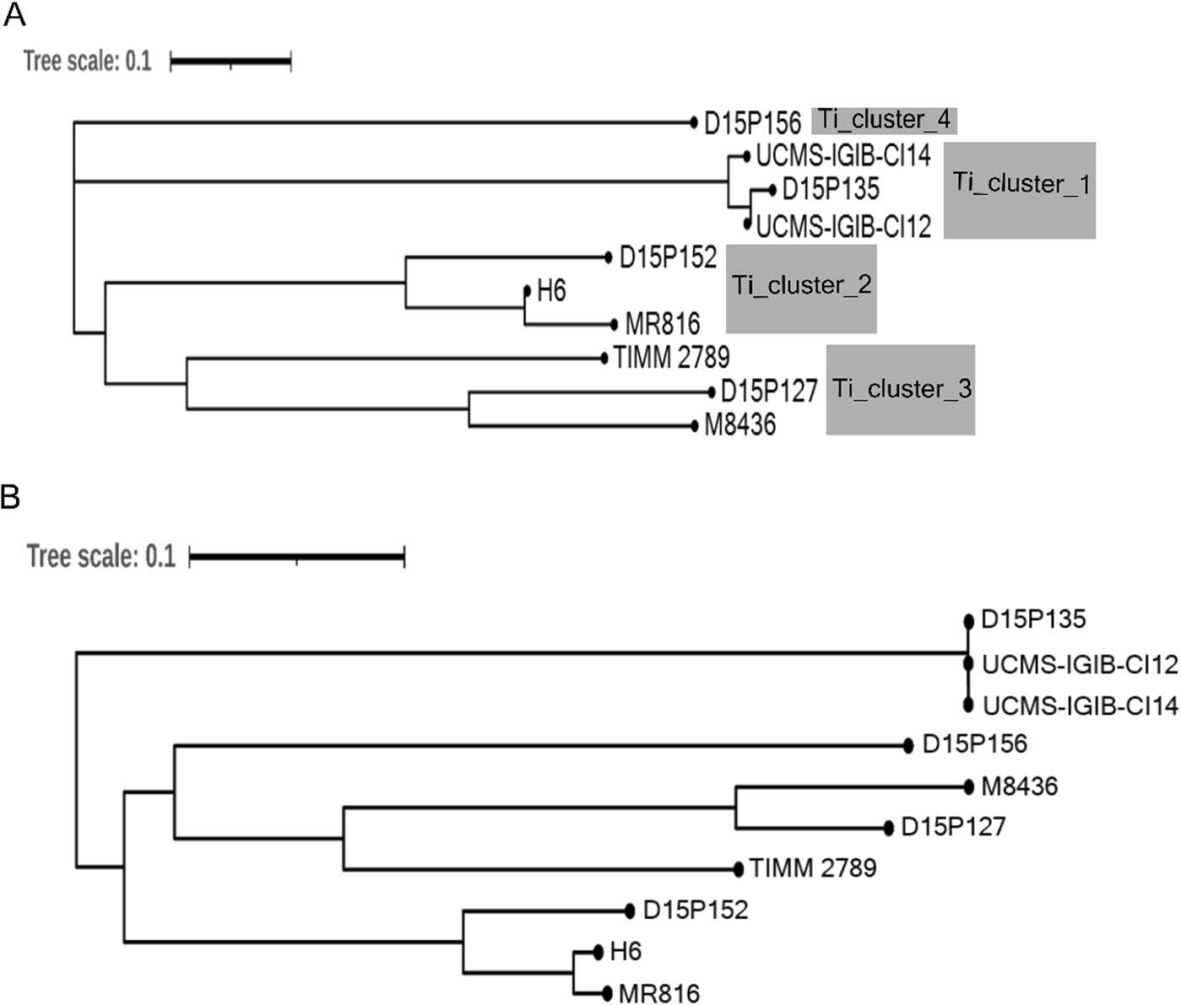
Genome relatedness of *T. mentagrophytes/ T. interdigitale* species complex using ANI and SNP-based phylogenomics analysis. **A** ANI-based and **B** SNP-based trees.

Whole genome sequences of *T. rubrum* (Table S2) and *T. mentagrophytes/ T. interdigitale* complex (Table S3) from India have become available in recent years. The overall relatedness of *T. rubrum* or *T. mentagrophytes/ T. interdigitale* complex strains from India with those from other countries was next carried out through ANI- and SNP-based phylogenomics. On the basis of pairwise nucleotide-level comparisons (ANI), *T. mentagrophytes/ T. interdigitale* complex strains formed four distinct clusters (Figs. 5a and S4). *T. mentagrophytes/ T. interdigitale* strains from India i.e. *T. mentagrophytes* D15P135 (D15P135), *T. indotineae* UCMS-IGIB-CI12 (UCMS-IGIB-CI12) and *T. indotineae* UCMS-IGIB-CI14 (UCMS-IGIB-CI14) are highly related to each other and together form Ti_cluster_1 (Fig. 5a). Two additional clusters with multiple members were observed in the ANI-tree comprising of *Trichophyton* sp. D15P152 (D15P152), *Trichophyton* sp. H6 (H6) and MR816 (Ti_cluster_2) or *T. mentagrophytes* D15P127 (D15P127), *T. mentagrophytes* TIMM2789 (TIMM2789) and *T. mentagrophytes* M8436 (M8436) (Ti_cluster_3). *T. mentagrophytes* D15P156 (D15P156) did not group with any of the other isolates and was annotated as Ti_cluster_4 (Fig. 5a).

A SNP-based phylogenetic tree for *T. mentagrophytes/ T. interdigitale* complex also formed four distinct clusters with the same members (Fig. 5b). When compared with the representative genome MR816, the estimated SNPs between members of each cluster were low as compared to members of other clusters. For instance, MR816 belongs to Ti_cluster_2 and revealed 5,961 SNPs with H6 and 28,768 SNPs with D15P152, both members of Ti_cluster_2. When MR816 was compared to other genomes, 73,764, 88,951 or 92,155 SNPs were identified for TIMM2789, D15P127 and M8436 (Ti_cluster_3) and > 95,000 SNPs with each member of Ti_cluster_1. However, all members of Ti_cluster_1 had < 1,000 SNPs between them, indicating very high genome relatedness with each other (Fig. 5b).

**Fig. 6:**
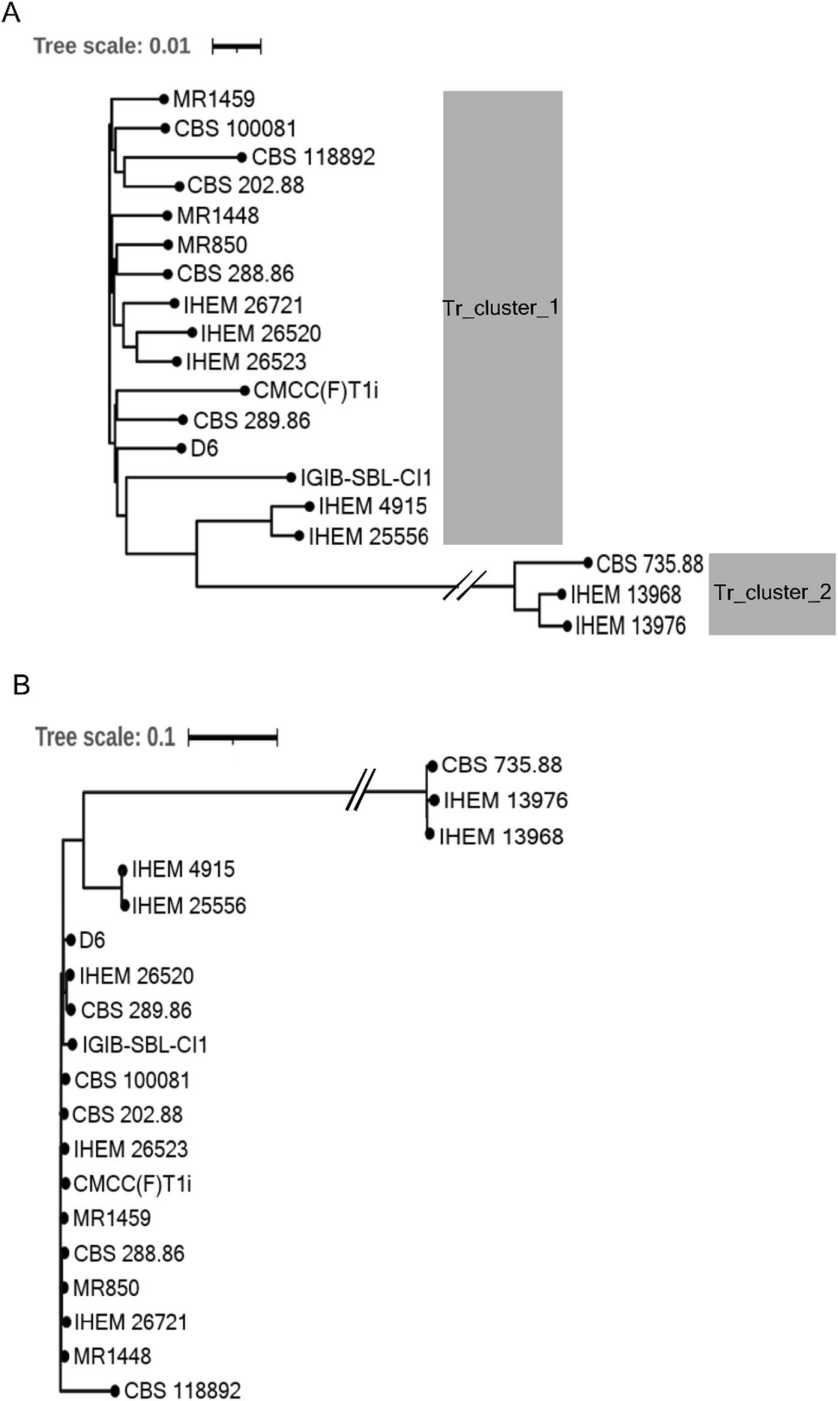
Genome relatedness of *T. rubrum* using ANI and SNP-based phylogenomics analysis. **A** ANI-based and **B** SNP-based trees

Overall relatedness of all the analyzed *T. rubrum* genomes was even higher with only two distinct clusters; a major cluster comprising of 16/19 T*. rubrum* strains (Tr_cluster_1) and a minor cluster comprising of 3/19 strains (Tr_cluster_2) (Figs. 6a and S5). All the three members of Tr_cluster_2 have recently been identified as *T. megninii* [43] and explains the separate cluster formed by these three isolates. The SNP-based phylogenetic tree also shows one major and one minor branch consisting of the same members as the ANI-tree (Fig. 6b). As expected, when compared with the representative genome CBS 118892 which belongs to Tr_cluster_1, members of Tr_cluster_1 were found to have lower number of SNPs (less than 8,406 SNPs for any member) than members of Tr_cluster_2 (more than 19,000 SNPs for each member).

### Sequence Analysis of Molecular Targets

With an increasing number of reports of poor response of dermatophytes to therapeutics in India over the last few years, sequence analysis of respective molecular targets, i.e. Erg1 (allylamines viz. terbinafine), Erg11 (azoles) and β-tubulin (griseofulvin) was next carried out for both *T. mentagrophytes/ T. interdigitale* complex and *T. rubrum* sequences against the respective reference strains (MR816 or CBS 118892).

Protein sequences of Erg1 of *T. mentagrophytes/ T. interdigitale* complex contained one or more mutations (Table 2A) in several members when compared to MR816. Mutations in all three members of Ti_cluster_1 (L393F in D15P135 and A448T in UCMS-IGIB-CI12 and UCMS-IGIB-CI14) and in two out of three members of Ti_cluster_3 (K276N and L419F in D15P127 and M8436) could be identified. No Erg1 mutation was identified in any of the members of Ti_cluster_2 or Ti_cluster_4. While none of the observed mutations seen in Erg1 in Ti_cluster_3 members are known to be associated with resistance to terbinafine, all the observed mutations identified in genotype VIII strains (Ti_cluster_1), are known to be associated with low or high levels of resistance to terbinafine, suggesting possible higher prevalence of drug resistance to terbinafine in dermatophytes from India [25, 37, 40].

**Table 2:** Mutations identified in Erg1, Erg11 and β-tubulin

Strain name	Erg1		Erg11		β-tubulin
A. *T. mentagrophytes*/ *T. interdigitale* species complex (against MR816)					
D15P135	L393F		K13E; D441G; R489K		–
UCMS-IGIB-CI12	A448T		K13E; R489K		–
UCMS-IGIB-CI14	A448T		K13E; R489K		del(Lys55-Ala56)
D15P156	–		K13E; Q264K		–
M8436	K276N; L419F		K13E; A231V		–
D15P127	K276N; L419F		K13E; D71N		^$^
TIMM2789	–		K13E; R489K		–
D15P152	–		–		^$^
H6	–		–		^$^
B. *T. rubrum* (against CBS 118892)					
CBS 735.88	–		–		–
IHEM 13976	–		–		–
IHEM 13968	–		–		–
IHEM 4915	–		–		–
IHEM 25556	–		–		–
D6	–		–		–
IHEM 26520	–		–		–
CBS 289.86	–		–		–
IGIB-SBL-CI1	L393F		–		–
CBS 100081	–		–		–
CBS 202.88	–		–		–
IHEM 26523	–		–		–
CMCC(F)T1i	–		–		–
MR1459	–		–		–
CBS 288.86	–		–		–
MR850	–		–		–
IHEM 26721	–		–		–
MR1448	–		–		–

^$^Partial length

Among the protein sequences of Erg11, again Ti_cluster_2 and Ti_cluster_4 sequences were identical to MR816 and did not harbor any mutations (Table 2A). However, one or more mutations were identified in members of all other clusters. None of these mutations in Erg11 though are known to be associated with azole resistance in any strain thus far [40]. β-tubulin, the target for griseofulvin, is a housekeeping protein required for structural maintenance of the cytoskeleton. β-tubulin exhibited higher level conservation among all *T. mentagrophytes/ T. interdigitale* complex members. Only UCMS-IGIB-CI14 contained a 2-residue deletion (Table 2A and Fig. S6) in the β-tubulin protein sequence. No griseofulvin resistance, however, has been reported for this strain [40].

Resonating with the higher similarity among *T. rubrum* genome sequences (> 99.79%), (Fig. S5), sequence analysis of molecular targets among *T. rubrum* strains also exhibited high level of similarity and no mutations were identified in examined Erg11 and β-tubulin (Table 2B). Erg1 sequences of *T. rubrum* genomes were also largely similar with the only India isolate, IGIB-SBL-CI1, harboring a terbinafine resistance-associated L393F mutation (Table 2B).

In summary, although the number of whole genome sequences of dermatophytes available from India is currently low for an unambiguous assessment, current analysis suggests a somewhat higher probability of resistance-associated mutations, especially to terbinafine, in *T. mentagrophytes/ T. interdigitale* complex Genotype VIII strains from India.

## Discussion

Dermatophytosis is one of the most common superficial infections of the skin affecting nearly one-fifth of the world population at any given time [5–7]. It is associated with significant morbidity and has substantial clinical consequences due to the chronic nature of infections and leads to a significant loss of Quality of life (QoL) [2, 3, 44]. In addition, recent studies have also highlighted the increasing incidences of drug resistance from several countries including India, Japan, the Middle East as well as Switzerland, Germany, Denmark in Europe, further compounding the problem [32, 37, 45–47]. A recent review comparing the antifungal susceptibility of terbinafine among *Trichophyton* spp. across the world indicated as many as 30% articles analyzed with reported change in MICs to terbinafine, pertained to India [48], emphasizing that India carries a significant burden of the emerging drug resistance epidemic. There is a congruent increase in reported number of cases including a possible change in causative agents to *T. mentagrophytes/ T. interdigitale* complex genotype VIII strains [21, 32], especially in the last decade [26]. However no comprehensive data is available for better estimations and overall assessment of prevalent pathogenic strains. We hence analyzed more than 160,000 cases by screening 1038 articles reporting dermatophytosis in India from 1939 to 2021 (Fig. S1) and thus enabling detailed epidemiological analysis over the investigated time period. Although dependent on hospital based studies reported in literature, they cover cases prevalent in and reported from all parts of the country (Fig. 1a-b) and emphasize the comprehensive nature of available data.

Based on literature mining of published studies and case reports, the first report indicating prevalence of dermatophytosis in India could be traced back to 1939, which was the analysis of tinea capitis cases in children [49]. Any details of dermatophytosis and prevalent agents prior to that are limited to medical records of local hospitals only. Only few publications were found upto 1960 and the first analyzed time period was taken as a broader set from 1939 to 1960. Due to the fewer initial reports, a slight numerical bias for studies with large sample sizes is likely from this period. For instance, a single large study of more than 800 patients indicates a significant proportion (33%) of infections is by *Epidermophyton* spp. between 1939 and 1960 [50]. With increased reporting and larger number of publications from multiple locations in subsequent years, any potential skew or bias due to reporting numbers is assumed to be normalized.

### A recent Shift in Dermatophyte Spectrum

The dermatophyte spectrum is not static and has been found to vary across different locations in the world as well as across different time periods [4, 11, 15, 16]. Our analysis of dermatophytosis in India in the last 80 y indicates that although the distribution of the most prevalent pathogen varies across the years, *T. rubrum* is the most common pathogen from 1939 to 2015 across all analyzed time periods (except between 1971 and1980) (Fig. 2). In the period 1971–1980, a high number of tinea capitis cases in children along with a higher proportion of its primary causative agent *T. violaceum* were observed [51–55]. Post 2015, there is a dramatic shift in dermatophyte spectrum with a two-fold increase in *T. mentagrophytes/ T. interdigitale* complex cases over 2011–2015 and a four-fold increase over 2001–2010, resulting in *T. mentagrophytes/ T. interdigitale* complex as the predominant pathogen responsible for > 65% of all cases between 2015 and 2021 in India (Fig. 2j).

While the sudden observed increase in dermatophyte infections (5746 KOH positive cases between 2016 and 2021, vs. 2494 between 2011 and 2015) is perplexing and perhaps could even be attributed to better awareness among both patients and doctors in reporting the disease in the 'social media'-driven modern world or to advances in technology for quicker and better identification, there is a concurrent overwhelming circumstantial increase of recalcitrant as well as drug resistance reports in India in the last 4–5 years [20, 22, 30, 37, 56, 57]. The shift in dermatophyte spectrum to *T. mentagrophytes/ T. interdigitale* complex does not appear to be driven by the observed drug resistant *T. mentagrophytes/ T. interdigitale* complex strains alone, as drug resistance has been reported in *T. rubrum* as well between 2016 and 2021 [25, 30].

### No Apparent Climatic Niche

Among all the states, Tamil Nadu (in South India) and Delhi (in North India) together account for nearly two-fifths of all culture-positive dermatophytosis cases (3332/8417) from 1939 to 2010 (Fig. 1a-b), suggesting continuous monitoring of dermatophyte infections in these two states. There are only sporadic dermatophytosis reports from other states upto 2010, followed by a dramatic increase in reports from multiple states with highly divergent climatic conditions (dry heat to coastal humid) (Fig. 1a). In addition, although large-scale therapeutic failure to antifungal agents (namely, terbinafine) was not reported upto 2018, the increased reports of dermatophytosis had become available prior to that from 2013 onwards (Fig. S1). It is hence provocative to suggest that the increased publications since 2013 are irrespective of an apparent region-specific climatic niche and represent the emergence of severity in both naive and recalcitrant cases from zones of multiple environments.

### Preponderance of Males

A preponderance of males towards dermatophyte infections is observed across most of the analyzed time periods in this study. While any reason for the gender bias is not apparent, we propose social reasons, i.e. more reporting by male patients or occupational hazards, i.e. more males getting manual-labor-associated occupations and related occupational injuries, making them further prone to infections, as possible reasons.

### High Concurrence of Phylogenetic and Phylogenomic Trees of *T. mentagrophytes/ T. interdigitale*

The changing dermatophyte spectrum led us to investigate the phylogenetic and phylogenomic profiles using available sequences in Genbank. *T. mentagrophytes/ T. interdigitale* complex has been divided into genotype I to IX, wherein Genotype VIII was recently reannotated as *T. indotineae* [32, 33]. Any *T. indotineae* annotations were hence considered along with *T. mentagrophytes/ T. interdigitale* Genotype VIII as per earlier annotation. The ITS-based tree that included reference strains of *T. mentagrophytes/ T. interdigitale* complex genotypes I to IX, indicated that more than 97% strains from India (i.e. 449/462) cluster with Genotype VIII while the remaining 13 strains cluster with genotype I/II/II*, genotype III* or genotype V. The classification of strains from India as a separate genotype VIII hence has overwhelming support and the small fraction of other genotypes coming through foreign travel as seen earlier for D15P135 [58] or zoonotic transfer [59, 60] cannot be discounted.

The ANI- or SNP-based phylogenomic trees for *T. mentagrophytes/ T. interdigitale* complex are in agreement with 18S rRNA-based phylogenetic tree as all strains with available whole genome sequences clustered in equivalent branches in the ANI- and SNP-trees. For instance, members of Ti_cluster_1 belong to the previously defined *T. mentagrophytes/ T. interdigitale* complex genotype VIII, while members of Ti_cluster_2 belong to the "cosmopolitan" genotype I/ II/ II*) [27, 28] (Fig. S7) reinforcing high concurrence of SNP-, ANI- and 18S-trees. All the three strains from India for which whole genome sequences are available, together cluster with genotype VIII in the 18S-phylogenetic tree (Fig. 3b) and as Ti_cluster_1 in the ANI-based phylogenomic tree (Fig. 5a). Ti_cluster_1 genomes are highly similar to each other with < 1000 SNPs between each other. The high similarity of *T. mentagrophytes*/ *T. interdigitale* complex genomes from India, with < 42 SNPs, was also indicated in another report recently [37], although the unavailability of these genomes limited their further phylogenomics assessment.

The high similarity of genome-wide features among available genomes was further confirmed through analysis of mutations in drug-response associated molecular targets (Table 2A, Table 2B) of each ANI-based cluster. Mutations associated with any drug resistance could be identified only towards terbinafine in Ti_cluster_1 but not in other clusters. We could identify a previously unreported L393F mutation in D15P135 (Table 2A). Although no drug sensitivity information is previously available for this strain, it is likely to be resistant to terbinafine based on this mutation. However, the sample size of three genomes is very small to assume that all genotype VIII strains would have poor drug sensitivity and additional genome sequences along with their drug sensitivity would help address this more precisely.

### H5*: a New Haplotype for *T. rubrum* Strains of India

18S rRNA-based and whole genome-based phylogenetic profiling has earlier revealed 12 distinct haplotype patterns for *T. rubrum* complex with *T. soudanese*, *T. violaceum*, *T. rubrum*, *T. megninii, T. kuryangei* and *T. yaoundei* as representative members of each haplotype, with *T. rubrum* primarily as haplotype_H5 (Fig. 3a). Any further population differentiation among each *Trichophyton rubrum* complex haplotypes was not possible due to high similarity among them [36, 42]. An ITS-based phylogenetic tree for all available 18S rRNA sequences of *T. rubrum* from India resulted in one additional sub-group within the haplotype_H5 members, which we have annotated as H5* (Fig. 3a). *T. rubrum* IGIB-SBL-CI1, the only *T. rubrum* strain from India for which whole genome sequence is available, is part of the H5 but not the H5* sub-group. Interestingly, all reference strains of H5_haplotype cluster with H5 rather than H5* strains. It is hence possible that H5* strains may have unique speciation and/or geographic specificity. However, in the absence of additional *T. rubrum* genome sequences from India, a possible sub-group formation of *T. rubrum* strains from India cannot be unambiguously explained.

ITS-based phylogenetic trees for *T. tonsurans* and *M. gypseum* were also generated, albeit with few sequences (Fig. 4a-b). For both these pathogens, one major phylogenetic cluster was formed, suggesting close relatedness among these strains as well.

### Sudden Emergence of Increasing Drug Resistance: Time to Revisit Therapy?

Our analysis of literature-based patterns of dermatophytosis in India over the last 80 y indicates *T. rubrum* to be the primary pathogen and *T. mentagrophytes*/ *T. interdigitale* complex as the second most prevalent pathogen upto 2015. There are no cases of reported drug resistance in India prior to 2016 although recalcitrant and difficult to treat *T. mentagrophytes*/ *T. interdigitale* complex cases were intermittently reported [56, 61, 62]. Post-2016, there is (a) a sudden increase in literature reports of dermatophytosis, (b) increase in drug resistance cases, and (c) change in dermatophyte spectrum to *T. mentagrophytes*/ *T. interdigitale* complex as the primary pathogen. Although it might be prudent to draw a firm conclusion through these associations, the circumstantial relatedness of terbinafine-resistance with the change in dermatophyte spectrum is overwhelming. However, this sudden change is intriguing. As one of the possible explanations for increasing *T. mentagrophytes*/ *T. interdigitale* infections in recent years, it is provocative to propose that a *T. mentagrophytes* zoophilic strain from pets or farm animals may have broken interspecies barriers and the sudden emergence of higher number of incidences and drug resistance are linked to anthropization and adaptation of the zoophilic strain. This hypothesis is not completely unfounded and animal-to human transmission crossing the interspecies barriers are often seen in dermatophytes [60, 63].

The search for possible answers and alternative explanations for higher recalcitrant cases is even more challenging as terbinafine-resistance has been reported in a few cases for *T. rubrum* as well [25, 30]. While mutations in the terbinafine target (Erg1 or squalene epoxidase) are the primary reason for drug resistance in both *T. mentagrophytes*/ *T. interdigitale* and *T. rubrum* [45, 64], additional factors, namely, improper dosage of drugs and steroid-misuse related induced change have also been reported in several recalcitrant cases [24, 26].

With the overwhelming association of the current dermatophyte spectrum in India with terbinafine-resistance, alternate therapies must hence be explored. Although identification of *T. mentagrophytes*/ *T. interdigitale* and *T. rubrum* by classical methods of KOH mounts or morphological identifications on growth media is easy and routinely followed, it does not differentiate between mutant or non-mutant strains (Fig. S8). Molecular methods and AFST to identify mutations in Erg1 and any change in MICs are available and also described in recent detailed reviews [65, 66], but they can often be challenging for untrained lab personnel and time-consuming, leading to considerable delay in treatment. Despite choice of terbinafine as the preferred fungicidal agent for a considerable amount of time, starting with a different class of antifungals doesn't seem uncalled for in light of the high drug resistance being observed and must be evaluated.

### Limitations

The current analysis is largely dependent on change in reporting status of dermatophytes in literature over the last few decades. In addition, the search for epidemiological data was limited by the keywords defined in the Methods section. Other literature formats viz. web-servers or books were not included due to limited access and to avoid repetition of data. Another limitation of this work is that since this is a hospital-based study, it may not reflect the true pattern in the community. However, despite the expected skew towards larger hospitals and dependency of such reports through treating doctors, the study is comprehensive as it covers reports from several tertiary care centres located in multiple locations having variable climatic conditions in the country.

Another potential limitation is that gender information was not available for all analyzed case studies. In addition, gender distribution was not usually provided for children and in some reports age-groups are provided in ranges of 5 or 10 y (e.g. 5–10y, 10–15y and so on or 0–10y 10–20y and so on). All these ranges, wherever available, were included as children, with the minor age-group being a significant proportion of that class. Nevertheless, 176/330 reports were available for assessing gender distribution, somewhat overcoming this limitation as well. Finally, the low number of available whole genome sequences for *T. mentagrophytes*/ *T. interdigitale* complex or *T. rubrum* limits phylogenomics studies and additional genomes may be required to further consolidate these studies.

## Conclusions

In conclusion, we have carried out a comprehensive analysis of all available literature pertaining to dermatophytosis in India over the last 80 years. We find that dermatophytosis is prevalent in all parts of the country despite variable climatic conditions in different regions with active reporting from all across the country. Overall pathogenic distribution indicates *T. rubrum* to be most prevalent pathogen upto 2015 with a sudden change in the dermatophyte spectrum towards *T. mentagrophytes*/ *T. interdigitale* complex post-2015. In addition, a concomitant increase of terbinafine resistance in both *T. rubrum* and *T. mentagrophytes*/ *T. interdigitale* complex is observed, suggesting it may be time to revisit antifungal therapy, especially in recalcitrant cases. Although whole genome sequence analysis showed that *T. mentagrophytes*/ *T. interdigitale* Genotype VIII strains from India have very high relatedness with low number of SNPs among each other, additional whole genome sequences of *Trichophyton* spp. from India would be required to help understand the sudden emergence of very high number of dermatophytosis cases and specific features of the prevalent stains in India. Finally, we feel the comprehensive literature mining and phylogenomics analysis of dermatophytosis carried out by us would help the medical and scientific community in making informed decisions and better disease management especially towards emerging resistance cases in India.

## Supplementary Information

Below is the link to the electronic supplementary material.

Supplementary file1 (DOCX 2584 KB)

Supplementary file2 (XLSX 45 KB)
