# Supplementary material for: Insights into Changing Dermatophyte Spectrum in India Through Analysis of Cumulative 161,245 Cases Between 1939 and 2021
Source: Mycopathologia. 2023 Mar 28;188(3):183–202. doi: 10.1007/s11046-023-00720-6 (PMC10307719; doi:10.1007/s11046-023-00720-6)
Supplement: Supplementary file 1 — Supplementary file1 (DOCX 2584 KB) [file 11046_2023_720_MOESM1_ESM.docx]

**Supplementary information**

**Insights into changing dermatophyte spectrum in India through analysis of cumulative 161,245 cases between 1939-2021**

Pawan Kumar^1, 2^, S. Ramachandran^1, 2^, Shukla Das^3^, SN Bhattacharya^4^ and Bhupesh Taneja^1, 2^*

^1^CSIR-Institute of Genomics and Integrative Biology, New Delhi, India; ^2^Academy of Scientific and Innovative Research, New Delhi, India; ^3^UCMS-GTB, Hospital, Dilshad Garden, Delhi. India; ^4^Dr. Babasaheb Ambedkar Medical College and Hospital, Delhi, India.

*Correspondence: btaneja@igib.res.in

**Table S1:** Complete list of 330 articles from 1939 to 2021 used in this study.

**Table S2:** Genomic features of *Trichophyton rubrum* genomes used in this study.

| **Strain name** | **Country** | **Genome accession no.** | **Genome size (Mb)** | **Coverage (x)** | **18S rRNA accession no.** | **Reference** |
| --- | --- | --- | --- | --- | --- | --- |
| CBS 118892 | Germany | ACPH00000000 | 22.53 | 8.19x | KT155731.1 | ^1,2^ |
| CBS 289.86 | Canada | AOKV00000000 | 23.05 | 173.0x | MF926368.1 | ^1^ |
| CBS 100081 | Canada | AOKT00000000 | 23.06 | 73.0x | AJ270792.1 | ^1^ |
| IHEM 13976 | Belgium | CAJUYM000000000 | 22.13 | 100.0x | Not available | ^3^ |
| IGIB-SBL-CI1 | India | JPGR00000000 | 22.52 | 30.0x | OL614127.1 | ^4^ |
| IHEM 13968 | Belgium | CAJUXX000000000 | 22.18 | 100.0x | Not available | ^3^ |
| IHEM 26721 | Belgium | CAJUYV000000000 | 22.25 | 100.0x | MK299041.1 | ^3^ |
| IHEM 26523 | Belgium | CAJUYS000000000 | 22.16 | 100.0x | MK298975.1 | ^3^ |
| IHEM 26520 | Belgium | CAJUYW000000000 | 22.47 | 100.0x | MK298972.1 | ^3^ |
| IHEM 4915 | Belgium | CAJUYL000000000 | 22.30 | 100.0x | Not available | ^3^ |
| IHEM 25556 | Belgium | CAJUYP000000000 | 22.14 | 100.0x | MK298945.1 | ^3^ |
| CMCC(F)T1i | China | LHPM00000000 | 22.30 | 150.0x | Not available | ^5^ |
| CBS 735.88 | Spain | JHQM00000000 | 22.63 | 55.0x | Z97994.1 | ^1^ |
| MR1459 | UK | AOLC00000000 | 23.16 | 109.0x | Not available | ^1^ |
| MR850 | UK | AOKR00000000 | 23.16 | 90.0x | Not available | ^1^ |
| D6 | Vietnam | AOLB00000000 | 23.12 | 109.0x | Not available | ^1^ |
| CBS 202.88 | Canada | AOKX00000000 | 23.11 | 70.0x | AJ270804.1 | ^1^ |
| MR1448 | UK | AOKZ00000000 | 23.12 | 174.0x | Not available | ^1^ |
| CBS 288.86 | Canada | AOKU00000000 | 23.12 | 167.0x | AJ270793.1 | ^1^ |

**Table S3:** Genomic features of *Trichophyton mentagrophytes* / *T. interdigitale* species complex genomes used in this study.

| **Strain name** | **Country** | **Genome accession no.** | **Genome size (Mb)** | **Coverage (x)** | **18S rRNA accession no.** | **Reference** |
| --- | --- | --- | --- | --- | --- | --- |
| MR816 | Germany | AOKY00000000 | 22.46 | 56.0x | Not available | ^1^ |
| H6 | Brazil | AOKS00000000 | 21.92 | 91.0x | DQ786661 | ^1^ |
| M8436 | Thailand | FUFL00000000 | 22.59 | 35.0x | KT253558 | ^6^ |
| UCMS-IGIB-CI12 | India | JAATJQ000000000 | 22.05 | 143.7x | MW600527 | ^7^ |
| UCMS-IGIB-CI14 | India | JAAQVJ000000000 | 22.04 | 136.3x | MW600653 | ^7^ |
| D15P127 | Russia | QQSR00000000 | 23.71 | 15.0x | MH708281 | ^8^ |
| D15P135 | India | QQSQ00000000 | 22.49 | 12.0x | KY761968 | ^8^ |
| D15P152 | Russia | QQSP00000000 | 23.11 | 5.0x | MH708282 | ^8^ |
| D15P156 | Moldova | QQSO00000000 | 23.23 | 12.0x | MH708283 | ^8^ |
| TIMM 2789 | Japan | BFBS00000000 | 24.06 | 160.0x | Not available | ^9^ |

**
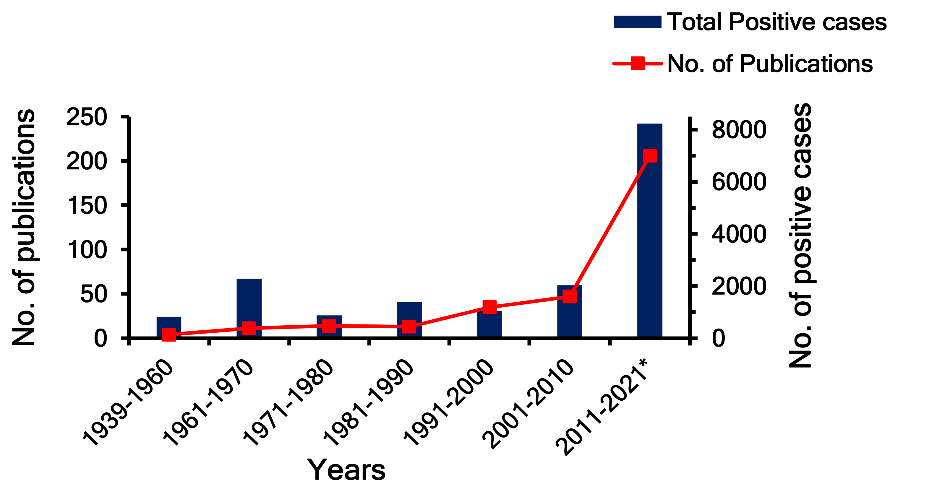
**

**Figure S1: Total reported cases of dermatophytosis from India across different decades.**

Decade-wise list of publications pertaining to dermatophytosis (red line) and KOH positive cases (blue bar) in India. 2011-2021 is marked with an "*" as data from 1-Jan-2021 to 30-Jun-2021 were included here in this and all subsequent analyses.

**Figure S2: Topological distribution**.

Decade-wise topographical distribution of dermatophyte infections. An overall plot for 1939-2021 is also shown**.**


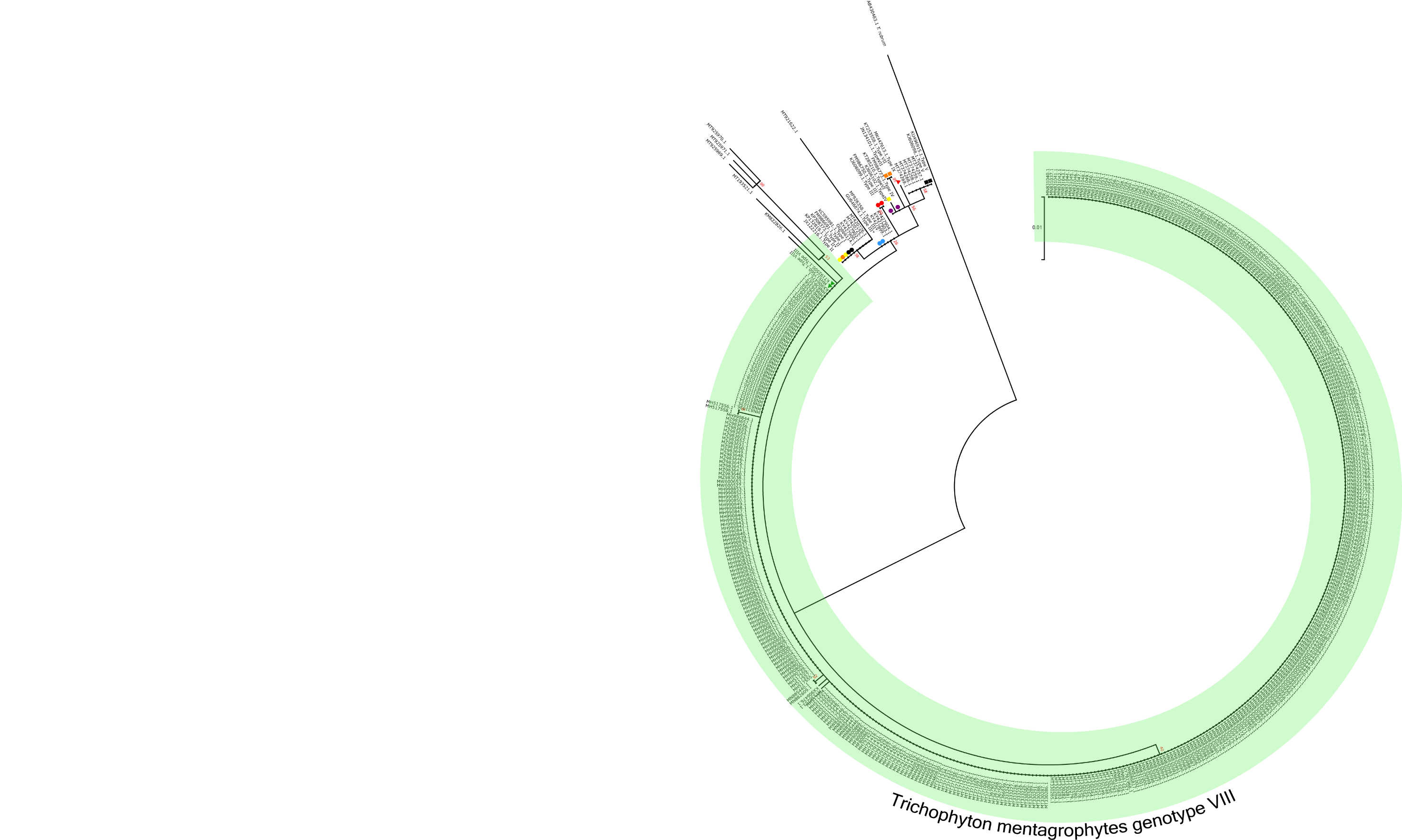


**Figure S3: 18S rRNA-based phylogenetic tree for *T. mentagrophytes/ T. interdigitale* isolates from India.**

An 18S rRNA-based maximum likelihood phylogenetic tree with 1000 bootstraps for 462 available isolates of *T. mentagrophytes/ T. interdigitale* species complex from India. Reference strains for genotypes I to IX are indicated in different colors and form separate clades. Majority of the strains from India (449/462) group as Genotype VIII (green). AB430483.1 (*T. rubrum*), MW683042.1 was used as an outlier for the phylogenetic tree.


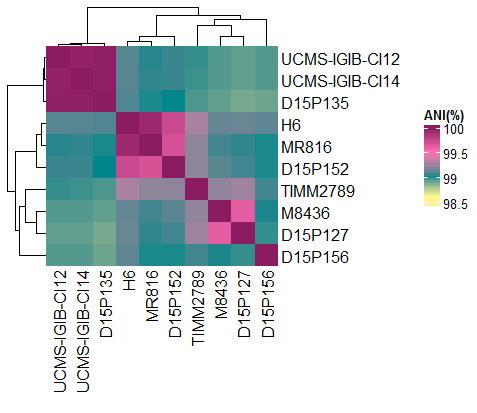


**Figure S4:** **ANI-based genome relatedness for *T. mentagrophytes/ T. interdigitale* species complex.**

Heatmap indicating relatedness of available genomes of *T. mentagrophytes/ T. interdigitale* species complex.

**Figure S5:** **ANI-based genome relatedness of *T. rubrum*.**

Heatmap indicating relatedness of available genomes of *T. rubrum*.

| D15P152 MREIVHLQTGQCGNQIGAAFWQTIAGEHGLDGSGHYTGSSDLQLERMNVYFNEAKASSKK 60  CBS118892 MREIVHLQTGQCGNQIGAAFWQTIAGEHGLDGSGHYTGSSDLQLERMNVYFNEA--SSKK 58  IGIB-SBL-CI1 MREIVHLQTGQCGNQIGAAFWQTIAGEHGLDGSGHYTGSSDLQLERMNVYFNEA--SSKK 58  UCMS-IGIB-CI14 MREIVHLQTGQCGNQIGAAFWQTIAGEHGLDGSGHYTGSSDLQLERMNVYFNEA--SSKK 58  M8436 MREIVHLQTGQCGNQIGAAFWQTIAGEHGLDGSGHYTGSSDLQLERMNVYFNEAKASSKK 60  MR816 MREIVHLQTGQCGNQIGAAFWQTIAGEHGLDGSGHYTGSSDLQLERMNVYFNEAKASSKK 60  UCMS-IGIB-CI12 MREIVHLQTGQCGNQIGAAFWQTIAGEHGLDGSGHYTGSSDLQLERMNVYFNEAKASSKK 60  D15P135 MREIVHLQTGQCGNQIGAAFWQTIAGEHGLDGSGHYTGSSDLQLERMNVYFNEAKASSKK 60  D15P156 MREIVHLQTGQCGNQIGAAFWQTIAGEHGLDGSGHYTGSSDLQLERMNVYFNEAKASSKK 60  TIMM2789 MREIVHLQTGQCGNQIGAAFWQTIAGEHGLDGSGHYTGSSDLQLERMNVYFNEAKASSKK 60  D15P127 MREIVHLQTGQCGNQIGAAFWQTIAGEHGLDGSGHYTGSSDLQLERMNVYFNEAKASSKK 60  ****************************************************** **** |
| --- |

**Figure S6: Sequence alignment of β-tubulin of *T. mentagrophytes/ T. interdigitale* species complex.**

Sequence alignment of β-tubulin of *T. mentagrophytes/ T. interdigitale* isolates highlighting the 2-residue deletion in UCMS-IGIB-CI14. No other sequence variation is observed in the remaining sequence length of β-tubulin of *T. mentagrophytes/ T. interdigitale* isolates. *T. rubrum* CBS 118892 and *T. rubrum* IGIB-SBL-CI1 sequences are also shown for comparison. The 2-residue deletion is seen in all *T. rubrum* sequences.

**Figure S7: 18S rRNA-based phylogenetic tree of *T. mentagrophytes/ T. interdigitale* species complex with reference Genotypes.**

An 18S rRNA-based maximum likelihood phylogenetic tree with 1000 bootstraps for *T. mentagrophytes/ T. interdigitale* isolates with available whole genome sequences in GenBank was generated along with reference sequences of each genotype as given by Nenoff et al [10]. The isolates cluster with closest related reference genotype (indicated as "Type N", where "N" is reference genotype number in Roman numerals) in respective clades.

**
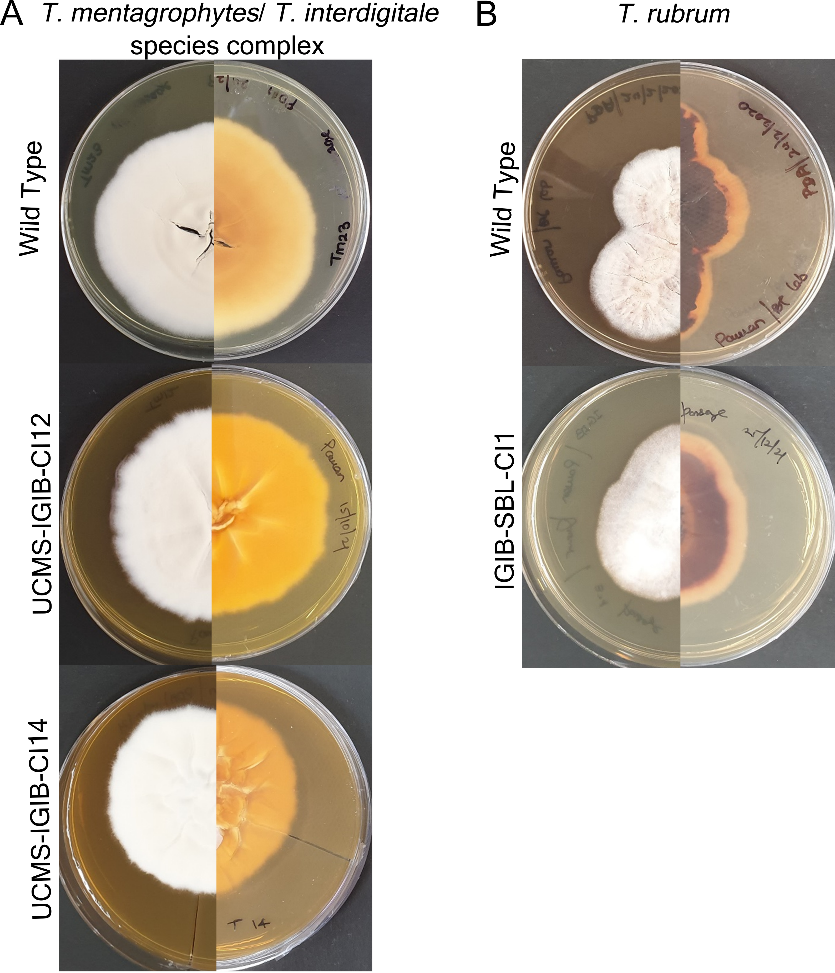
**

**Figure S8: Characteristic growth of clinical strains on growth media.**

Phenotypic traits of clinical isolates for (A) *T. mentagrophytes/ T. interdigitale* species complex, obverse and reverse and (B) *T. rubrum*, obverse and reverse, show different characteristic profiles for the two species. However, growth of *erg1* mutants is indistinguishable from respective non-mutant clinical strains (labeled as wild type)*.*

**References**

1. Persinoti, G. F. et al. Whole-Genome Analysis Illustrates Global Clonal Population Structure of the Ubiquitous Dermatophyte Pathogen Trichophyton rubrum. Genetics. 2018;208:1657–1669.

2. Martinez, D. A. et al. Comparative Genome Analysis of Trichophyton rubrum and Related Dermatophytes Reveals Candidate Genes Involved in Infection. mBio. 2012;3: e00259-12.

3. Cornet, L. et al. The taxonomy of the Trichophyton rubrum complex: a phylogenomic approach. Microb Genomics. 2021;7: 000707.

4. Latka, C. et al. Genome sequence of a clinical isolate of dermatophyte, Trichophyton rubrum from India. FEMS Microbiol. Lett. 2015;362:fnv039.

5. Zhan, P., de Hoog, S. & Liu, W. Draft Genome Sequences of Trichophyton rubrum CMCC(F)T 1i and Trichophyton violaceum CMCC(F)T 3l by Illumina 2000 and Pacific Biosciences. Genome Announc. 2017;5:e00920-17.

6. Gallo, J. G., Woods, M., Graham, R. M. & Jennison, A. V. A severe transmissible Majocchi’s granuloma in an immunocompetent returned traveler. Med. Mycol. Case Rep. 2017;18:5–7.

7. Kumar, P. et al. Whole genome sequences of two Trichophyton indotineae clinical isolates from India emerging as threats during therapeutic treatment of dermatophytosis. 3 Biotech. 2021;11:402.

8. Pchelin, I. M. et al. Species boundaries in the Trichophyton mentagrophytes / T. interdigitale species complex. Med. Mycol. 2019;57:781–789.

9. Alshahni, M. M. et al. Insight into the draft whole-genome sequence of the dermatophyte Arthroderma vanbreuseghemii. Sci. Rep. 2018;8:15127.

10. Nenoff P, Verma SB, Uhrlaß S, Burmester A, Gräser Y. A clarion call for preventing taxonomical errors of dermatophytes using the example of the novel Trichophyton mentagrophytes genotype VIII uniformly isolated in the Indian epidemic of superficial dermatophytosis. Mycoses. 2019;62:6–10.
